# 

**Published:** 1885-12

**Authors:** 


WHOLE NUMBER CCLXXX. DECEMBER, 1885. NUMBER 5. VOLUME XXV.

### THE BUFFALO Medical and Surgical Journal

LIBRARY INDEXED S.G.O.

ESTABLISHED IN YEAR 1844.

EDITORS:

THOS. LOTHROP, M. D. A. R. DAVIDSON, M. D.

ASSOCIATE EDITORS:

FLOYD S. CREGO, M. D. CARLTON C. FREDERICK, M. D.
FRED. R. CAMPBELL, M. D. HERBERT MICKLE, M. D.

Entered at the Post-Office at Buffalo, N. Y., as Second-class Mail Matter.

